# Txndc9 Is Required for Meiotic Maturation of Mouse Oocytes

**DOI:** 10.1155/2017/6265890

**Published:** 2017-05-24

**Authors:** Fanhua Ma, Liming Hou, Liguo Yang

**Affiliations:** Key Laboratory of Agriculture Animal Genetics, Breeding and Reproduction, College of Animal Science, Huazhong Agricultural University, Wuhan 430070, China

## Abstract

Txndc9 (thioredoxin domain containing protein 9) has been shown to be involved in mammalian mitosis; however, its function in mammalian oocyte meiosis remains unclear. In this study, we initially found that Txndc9 is expressed during meiotic maturation of mouse oocytes and higher expression of Txndc9 mRNA and protein occurred in germinal vesicle (GV) stage. By using confocal scanning, we observed that Txndc9 localized at both nucleus and cytoplasm, especially at spindle microtubules. Specific depletion of Txndc9 by siRNA in mouse oocyte resulted in decreasing the rate of first polar body extrusion and increasing abnormal spindle assemble. Moreover, knockdown of Txndc9 in germinal vesicle (GV) stage oocytes led to higher level of reactive oxygen species (ROS) and lower level of antioxidant glutathione (GSH) as compared with control oocytes, which indicated that Txndc9 may be involved in mediating the redox balance. In summary, our results demonstrated that Txndc9 is crucial for mouse oocyte maturation by regulating spindle assembly, polar body extrusion, and redox status.

## 1. Introduction

In vitro maturation (IVM) of oocytes as a very valuable artificial reproductive technology plays an important role in the fields of reproductive and developmental biology [1]. During mammalian oocytes growth and maturation, both nucleus and cytoplasm maturation events are coordinated, including resumption of meiosis, proper spindle assembly, and polar body extrusion [2]. During these processes, rearrangement of organelles, cytoskeletal microtubules, and associated motor proteins such as dynein, dynactin, and kinesin participated [3]. Orderly meiosis in oocytes needs accurate control of spindle and chromosome movement. Microtubule assembly and dynamics of microtubule polymerization require many proteins, which are crucial for correct and efficient formation of tubulin heterodimer; afterwards a wide array of binding proteins and modifying enzymes further regulate microtubule function [4]. Any error in this process can result in the generation of aneuploid eggs. Fertilization of aneuploid eggs in humans is a major cause of pregnancy loss and infertility [5].

Txndc9 (thioredoxin domain containing protein 9) is required for microtubule formation and microtubule-dependent steps of cell division in* Arabidopsis* [6]. Txndc9 binds G protein and participates in signal transduction of G protein modulation and also the regulation of tubulin and actin function in yeast [7],* Caenorhabditis elegans* [8], and mammalian cells [9]. Overexpression of Txndc9 promotes an imbalance of *α*- and *β*-tubulin, microtubule disassembly, and cell death and disturbs the mitosis process [9]. Txndc9 is also named PhLP3 (phosducin-like family of proteins 3), which is a CCT-binding protein [10–13]. In eukaryotes, the chaperonin containing TCP-1 (CCT, also termed TRiC or c-cpn) is required for folding nascent actin and tubulin [14] and newly synthesized proteins [15]. Study has shown that Txndc9 modulates CCT-mediated actin and tubulin folding via forming ternary complexes and plays a role in establishing functional cytoskeleton [10]. However, whether Txndc9 is required for tubulin assembly during mouse oocyte maturation remains unclear.

It is well known that tubulin disulfide formation and microtubule assembly are regulated by reactive oxygen species (ROS) or the thioredoxin reductase system [16, 17] and optimal repair of tubulin disulfides required thioredoxin [16]. Although Txndc9 is a member of TRX-domain family, it is still not clear whether Txndc9 is required for balance the redox during mouse oocyte development.

Moreover, one previous study has shown that Txndc9 is expressed in the mouse oocytes by RNA-seq [18], but its function during the maturation of mouse oocyte remains unclear. Here we investigated its function and found that depletion of Txndc9 by siRNA microinjection disrupted the maturity of mouse oocyte into MII stage, led to abnormal tubulin assembly, increased reactive oxygen species (ROS) level, and decreased antioxidant glutathione (GSH) level. Our results suggested the Txndc9 is essential for mouse oocyte maturation, by regulating microtubule assembly and oxidation balance.

## 2. Materials and Methods

### 2.1. Oocyte Collection and Culture

All animal experiments were carried out according to the Ethical Committee of the Hubei Research Center of Experimental Animals (Approval ID: SCXK (Hubei) 2008_0005). Kunming mice (3–6 weeks old) were obtained from the Center of Laboratory Animals of Hubei Province (Wuhan, China). The mice were sacrificed by cervical dislocation. Germinal vesicle (GV) oocytes were collected from their ovaries and were cultured in M16 medium (Sigma) covered with mineral oil for 0, 4, 8, 9.5, or 14 h in vitro, which represents the time points when mouse oocytes reached germinal vesicle (GV), germinal vesicle breakdown (GVBD), Metaphase I (MI), Anaphase/Telophase I (ATI), and Metaphase II (MII) stages, respectively. All cultures were carried out at 37°C in a humidified atmosphere of 5% CO_2_.

### 2.2. Microinjection

Approximately 5–10 pl of 25 *µ*M Txndc9 siRNAs (5′-GCGAGAGAGACUUCUUUCATT-3′) or control nonspecific siRNAs was microinjected into the cytoplasm of fully grown GV oocytes. After injection, the oocytes were arrested at the GV stage by supplemented with 5.0 *µ*M milrinone for 24 h in M2 medium, and after that, the oocytes were washed three times with milrinone-free fresh M16 medium. For antibody injection, 5–10 pl of Txndc9 antibody (Sigma) or negative anti-rabbit IgG was microinjected and then oocytes were cultured in M2 medium containing 5.0 *μ*M milrinone for 30 min. After washing three times in milrinone-free fresh M16 medium, oocytes were transferred to fresh M16 medium. Then the oocytes were harvested or continued to be cultured in fresh M16 medium covered with mineral oil to assigned time point at 37°C in an atmosphere of 5% CO_2_ in air.

### 2.3. Real-Time Quantitative PCR Analysis

Total RNA from 150 oocytes in each group was extracted by using RNeasy Plus Micro Kit (Qiagen, 74034 Germany). In accordance with the manufacturer's protocol, 14 *µ*l total RNA was carried out for Reverse Transcription (RT) reaction using the RevertAidTM First Strand cDNA Synthesis Kit (Fermentas, Canada) according to the manufacturer's protocol. Real-time quantification PCR was conducted on a CFX96 real-time PCR detection system (Bio-Rad, USA), by using SYBR® Premix Ex TaqTM II (TaKaRa, DRR081A, Japan). Glyceraldehyde-3-phosphate dehydrogenase (GAPDH) was used as a control gene to correct cDNA level of samples. The primers used were as follows: Txndc9: 5′-AGCAGCAGAAACAAGAGT-3′ (forward); 5′-CTGTAGAAATGGCAAACC-3′ (reserve) and GAPDH: 5′-GCCTTCCGTGTTCCTA-3′ (forward); 5′-AGACAACCTGGTCCTCA-3′ (reserve). Relative gene expression levels were calculated by using 2^−ΔΔC_T_^ method [19].

### 2.4. Western Blot Analysis

Samples containing at least 150 oocytes at assigned stages of meiosis were collected in 2x SDS loading buffer for Western blot analysis. Then samples were heated for 5 min at 100°C. After separation by SDS-PAGE, proteins were transferred to polyvinylidene fluoride (PVDF) membrane. After transfer, the membranes were blocked in 5% skim milk for 1 h, followed by overnight incubation at 4°C with polyclonal rabbit anti-Txndc9 (Sigma, HPA031845), anti-*β*-tubulin (CST, 2146), and anti-*α*-tubulin (CST, 2144). After three washes for 10 min each in TBST, the membranes were incubated for 1 h at 37°C with 1 : 2000 diluted horseradish peroxidase- (HRP-) conjugated goat anti-rabbit or anti-mouse IgG antibody and washed three times with TBST. Finally, membranes were developed using the ECL Western blotting detection system.

### 2.5. Immunofluorescence Staining and Confocal Microscopy

Microinjected oocytes were collected for immunofluorescence staining after specific durations of in vitro culture according to the previously described methods with some modification [20]. Oocytes were fixed in 4% paraformaldehyde in phosphate-buffered saline (PBS) for 30 min at room temperature. After permeabilization with 0.5% Triton X-100 in PBS at room temperature for 30 min, oocytes were incubated overnight at 4°C with polyclonal rabbit anti-Txndc9 (1 : 100, Sigma) diluted in PBS containing 2% BSA. After rinsing three times in wash buffer (PBS containing 0.1% Tween-20 and 0.01% Triton X-100) for 5 min each, the oocytes were further incubated in Goat Anti-Rabbit FITC (1 : 100) or Cy3-conjugated anti-rabbit IgG antibody (1 : 100) according to the host of the primary antibody. After three times of washes, oocytes were incubated with Anti-*α*-Tubulin−FITC antibody (1 : 100, sigma) diluted in PBS containing 2% BSA. Then, oocytes were then stained with 4,6-diamidino-2-phenylindole (DAPI; 10 *µ*g/ml in PBS) to visualize nucleus. After that, samples were mounted on glass slides and captured using confocal laser scanning microscope (Zeiss LSM 510 META, Carl Zeiss Imaging, A1, Germany) equipped with a Plan-Apochromat 63x/1.4 oil DIC objective with similar exposure adjustments. At least 10–15 oocytes were captured for each group. Image J software (National Institutes of Health, USA) was used to assess the immunostaining signal intensity. All images were taken under the same conditions, such as magnification, intensity of excitation light, gain, and offset.

### 2.6. Intracellular Reactive Oxygen Species (ROS) Level

In order to determine the quantities of ROS produced by the oocytes, oocytes were measured by nonionized 2,7-dichlorodihydrofluorescein diacetate (DCFH-DA), a nonfluorescent probe, which is converted to the sensitive fluorescent compound 2,7-dichlorofluorescein (DCF) when oxidized by intracellular ROS [21]. DCF is highly reactive with hydrogen peroxide and has been used in measuring the ROS generation in mammalian cells [22]. Microinjected GV and MII oocytes were exposed to 10 *μ*M DCFH-DA in vitro wash media containing 3 mg/mL of FAF-BSA for 30 min. Oocyte fluorescence intensity was measured at excitation and emission wavelengths of 500 nm and 529 nm, respectively. Images were analyzed by ImageJ software (National Institutes of Health, USA). The arbitrary units and relative fluorescence units (RFU) were based directly on fluorescence intensity. Three independent experiments were performed and 10–15 oocytes were examined per treatment group per replicate.

### 2.7. Measurement of Reduced Glutathione

We used monochlorobimane (MCB, Molecular Probes), a membrane-permeable fluorescence probe, as a sensitive and specific probe to determine intracellular reduced glutathione (GSH) content [23, 24]. Following Txndc9 siRNA injection and 0 h/14 h IVM, GV and MII stages oocytes were exposed to 100 *μ*M MCB in vitro wash medium containing 3 mg/mL FAF-BSA for 30 min. Excitation and emission wavelengths of 458 nm and 475 nm, respectively, were used to detect the fluorescent GSH-bimane adduct. Laser power and photomultiplier settings were kept constant for all experiments. Images were captured and oocyte fluorescence intensities were measured and analyzed using ImageJ software (National Institutes of Health, USA). Three independent experiments were performed using 10–15 oocytes per treatment group per replicate.

### 2.8. Statistical Analysis

At least three independent replicates were performed for each experiment and the data were expressed as means ± SD. Statistical comparisons were made by Student's *t*-test. A *p* value of <0.05 was considered significant.

## 3. Results

### 3.1. Expression and Localization of Txndc9 during Mouse Oocyte Meiotic Maturation

**Figure 1:**
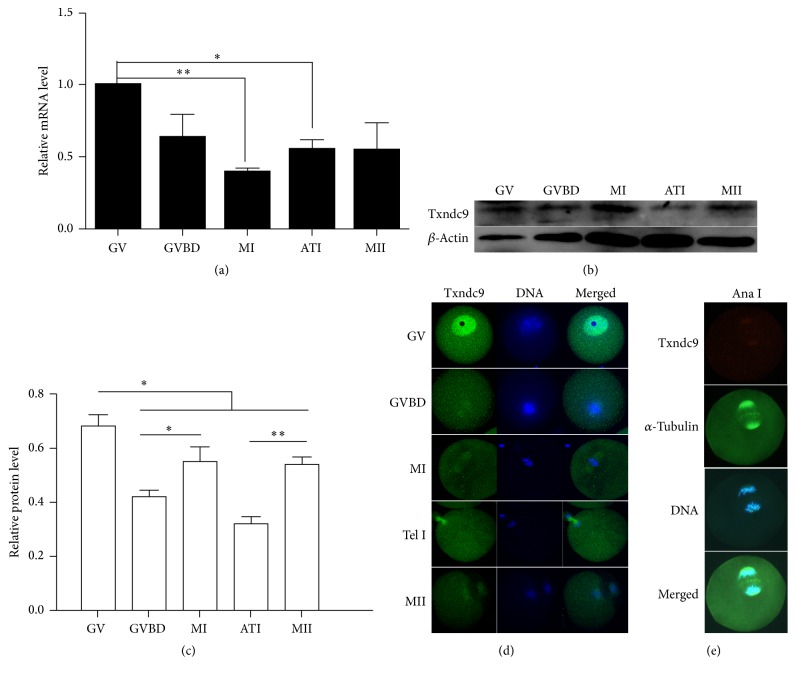
Expression and localization of Txndc9 during mouse oocyte meiosis maturation. (a) The mRNA level of Txndc9 during mouse oocyte meiosis maturation, GAPDH as the reference gene. (b) Txndc9 protein expression was detected by Western blot in GV, GVBD, MI, ATI, and MII stages and the relative protein level (Txndc9/*β*-actin) was analyzed and shown in (c). In (a) and (c), data are means ± SD from three independent experiments. ^*∗*^*p* < 0.05, ^*∗∗*^*p* < 0.01. (d) Subcellular localization of Txndc9 during mouse oocyte maturation in GV, GVBD, MI, ATI, and MII stages. Green: Txndc9; Blue: DAPI. (e) Oocytes were costained with anti-Txndc9 and anti-*α*-tubulin antibodies in AnaI stage. Green: *α*-tubulin; red: Txndc9; blue: DAPI.

We initially investigated the expression level of Txndc9 during mouse oocyte meiotic maturation. Germinal vesicle (GV), germinal vesicle breakdown (GVBD), Metaphase I (MI), Anaphase/Telophase I (ATI), and Metaphase II (MII) stages mouse oocytes were collected after being cultured in M16 medium for 0, 4, 8, 9.5, or 14 h, respectively. RT-qPCR result indicated that Txndc9 mRNA is expressed from GV to MII stage and the highest Txndc9 mRNA level occurred at GV stage (Figure 1(a)). Western blot result also showed that Txndc9 protein is expressed during oocyte maturation (Figure 1(b)) and GV stage has the highest Txndc9 protein level compared with other stages (Figure 1(c)).

Using immunofluorescent staining, we detected the subcellular location of Txndc9 at different stage of meiotic maturation. Txndc9 was predominantly accumulated in the nucleus at GV stage, appeared around chromosomes at GVBD stage, and localized at spindle microtubules from MI to the MII stage (Figure 1(d)). Meanwhile, we also detected the Txndc9 signal in the cytoplasm. Moreover, costaining of Txndc9 and *α*-tubulin at AnaI (anaphase) further confirmed the colocalization of Txndc9 and spindle microtubules (Figure 1(e)).

### 3.2. Txndc9 Depletion Affects Maturational Progression of Mouse Oocyte

**Figure 2:**
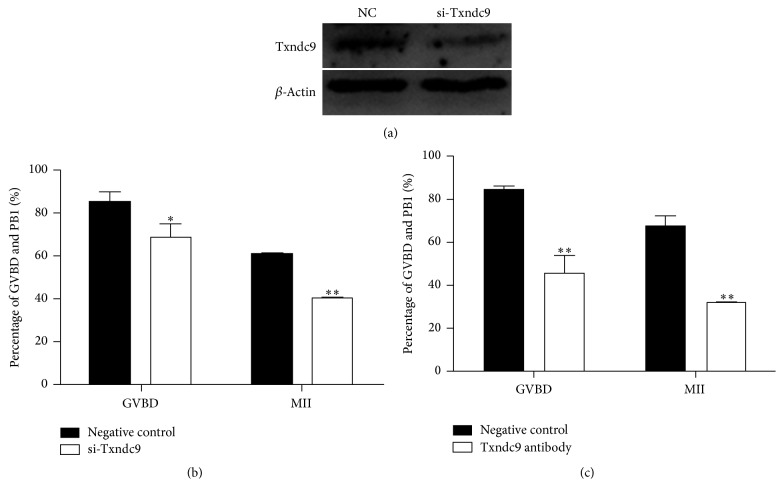
Effect of Txndc9 depletion on mouse oocyte meiotic maturation. (a) Knockdown of Txndc9 was evaluated by Western blot and *β*-actin as a loading control. (b) The development rate of oocytes in GVBD and MII stages after injecting si-Txndc9 or nonspecific siRNA (negative control). (c) Immunodepletion of Txndc9 by antibody injection decreased the development rate of oocytes reaching GVBD and MII stages. Data are means ± SD from three independent experiments in (b) and (c); ^*∗*^*p* < 0.05, ^*∗∗*^*p* < 0.01.

To investigate the potential roles of Txndc9 during mouse oocyte maturation, we injected the siRNA against Txndc9 into the cytoplasm of GV stage oocytes for 24 h in M2 medium, supplemented with 5.0 uM milrinone to block the development of GV stage oocytes. Then oocytes continued to be cultured for 4 h or 14 h in milrinone-free M16 medium to reach GVBD or MII stage, respectively. The knockdown efficiency was confirmed by Western blot (Figure 2(a)). Depletion of Txndc9 by injection of siRNA significantly decreased the oocyte developmental rate reaching GVBD (68.99 ± 6.25% versus 85.07%  ±  4.92%, *p* < 0.05) and MII stages (40.29 ± 1.50% versus 60.68%  ±  1.13%, *p* < 0.05) as compared with nonspecific siRNA control (Figure 2(b)). These results were not due to the off-target effect of siRNA-mediated knockdown, as we observed the similar results using another individual siRNA against Txndc9 (data not shown). We also injected anti-Txndc9 antibody into the cytoplasm of GV stage oocytes for 30 min in M2 medium supplemented with 5.0 *µ*M milrinone, and then we evaluated the oocyte development rate at GVBD and MII stages after being cultured for 4 h and 14 h in milrinone-free M16 medium, respectively, the results were consistent with our siRNA injection experiment, and we found that immunodepletion of Txndc9 dramatically reduced the mouse oocytes reaching GVBD (45.14 ± 8.16% versus 83.8 ± 1.97%, *p* < 0.05) and MII (30.75 ± 0.61% versus 66.6 ± 5.30%, *p* < 0.05) stage as compared to IgG control group (Figure 2(c)).

### 3.3. Txndc9 Is Critical for Spindle Assembly during Meiosis I

**Figure 3:**
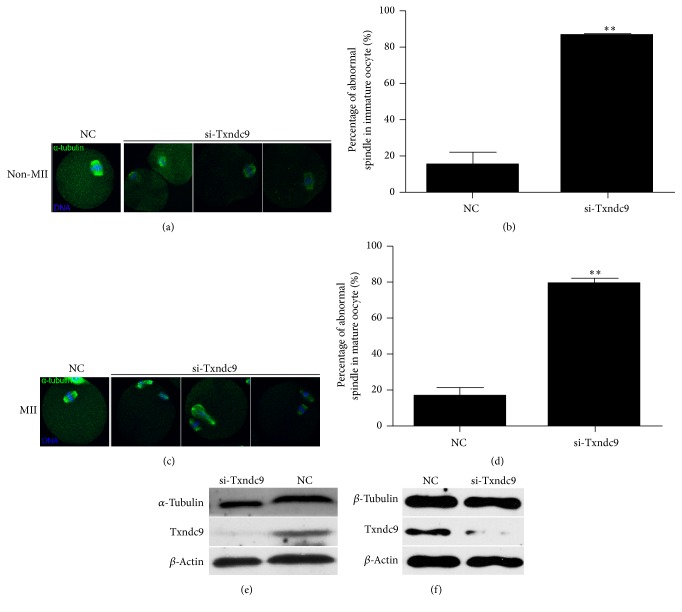
Txndc9 knockdown resulted in aberrant spindle morphology in immature and mature mouse oocytes. Representative confocal images showed the spindle morphology and chromosome alignment of immature oocytes (a) and mature MII oocytes (c) by immunostaining with *α*-tubulin to visualize spindle (green) and nucleus (DAPI, blue). Quantification of negative control and Txndc9-siRNA oocytes with spindle/chromosome defects in immature (b) and mature (d) oocytes, and values are shown as means ± SD; ^*∗*^*p* < 0.05, ^*∗∗*^*p* < 0.01. Western blots showed the protein level of *α*-tubulin (e) and *β*-tubulin (f) in si-Txndc9 and negative control (NC) group.

To further explore the reasons causing the failure of oocyte maturation after depletion of Txndc9. Following injection of si-Txndc9 and nonspecific siRNA, GV stage oocytes were cultured for 14 h in milrinone-free M16 medium, and then we evaluated chromosome alignment and spindles assembling status in immature (non-MII) and mature MII oocytes (MII) (Figures 3(a) and 3(c)). We found that most of immature oocytes (non-MII) in control group (NC) had their spindles migrated to the cortex with normal morphology; however, a large proportion of oocytes subjected with Txndc9 siRNA injection displayed chromosome misalignment and aberrant spindles (Figure 3(a)). The number of immature oocytes with abnormal spindles was significantly higher in Txndc9 knockdown than control group (86.61%  ±  1.26% versus 15.00%  ±  7.07%; *p* < 0.05) (Figure 3(b)). For the mature MII oocytes, a large proportion of Txndc9 depleted oocytes displayed chromosome misalignment and aberrant spindle as compared with control mature oocytes with normal morphology (Figure 3(c)). The number of mature MII oocytes with abnormal spindles in si-Txndc9 group was significantly higher than that in control group (79.20 ± 2.93% versus. 16.67 ± 4.71%; *p* < 0.05) (Figure 3(d)).

We further detected the protein expression level of *α*-tubulin and *β*-tubulin in GV stage oocytes after siRNA injection. There is no significant difference between Txndc9 knockdown and control groups (Figures 3(e) and 3(f)). These results indicated that the failure of spindle formation and polar body extrusion after loss of Txndc9 is due to abnormal assembling or folding of tubulin, rather than the protein expression change of tubulin.

### 3.4. Txndc9 Is Essential for the Balance of Redox during Meiosis I

Txndc9 is one of the members of the thioredoxin (Trx) family. It encodes a protein with 226 amino acids and contains a TRX-like domain. Txndc9 does not have the typical redox active CXXC motif; however, some of Trx-fold superfamily proteins possess an atypical redox active site [Thr-X1-X2-Cys] [25]. We found that Txndc9 also has an atypical redox active site [Thr-Phe-Arg-Cys] (TFRC), which suggested its potential function of redox balance.

**Figure 4:**
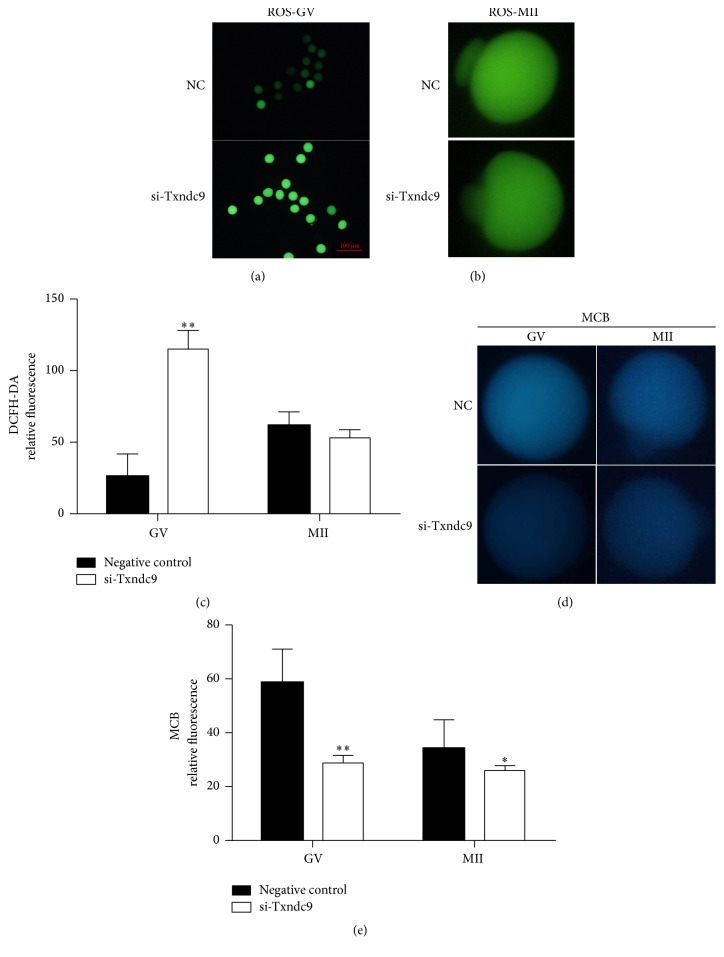
Effect of Txndc9 depletion on ROS and GSH levels during mouse oocyte maturation. Representative images of intracellular ROS production using DCFH-DA in GV (a) and MII (b) stage oocytes in negative control (NC) and si-Txndc9 groups; (c) the relative level of ROS was quantified in negative control and Txndc9-siRNA. Representative fluorescence images (d) and relative level monochlorobimane (MCB) (e) in GV and MII stage oocytes after loss of Txndc9. Data represents means ± SD; ^*∗*^*p* < 0.05, ^*∗∗*^*p* < 0.01.

Next, we measured the ROS and GSH levels of GV stage oocytes with or without microinjection of si-Txndc9 after 24 h supplement with milrinone (Figure 4(a)) and also the oocytes reached MII stage after microinjection of si-Txndc9 followed by 14 h in vitro culture without milrinone (Figure 4(b)). The level of oocytes' ROS production was measured by DCFH-DA fluorescence, and loss of Txndc9 significantly increased DCFH-DA fluorescence in GV stage (Figures 4(a) and 4(c), left bars; *p* < 0.05), but not in MII stage (Figures 4(b) and 4(c), right bars) as compared with negative control (NC). In order to evaluate the reduction-oxidation ability of mouse oocytes, antioxidant reduced glutathione (GSH) was measured using monochlorobimane (MCB), which can form a fluorescent adduct following an enzyme catalyzed reaction with GSH [26]. The results indicated that injection of siRNA against Txndc9 significantly decreased MCB fluorescence in GV and MII stage as compared to control (Figures 4(d) and 4(e); *p* < 0.05).

## 4. Discussion

Little is known about the biological functions of Txndc9 during mouse oocyte meiosis. Prior to exploring the function of Txndc9 in the process of mouse oocyte maturation, we first confirmed its expression in mouse oocytes using WB and qPCR. These results were consistent with a previous study that Txndc9 is expressed in the stages of GV and MII mouse oocytes by RNA-seq [18]. Our study showed that Txndc9 is highly expressed in oocyte GV stage. We assumed that Txndc9 plays important role in the process of meiosis and oocyte maturation.

Txndc9 emerged in both nucleus and cytoplasm and it localized to centrosomes and midbody during cytokinesis in Hela cells [8]. There is no obvious centrosome in mouse oocytes and we observed that Txndc9 existed in both nucleus and cytoplasm at GV stage, accumulated around chromosomes, and localized at spindle microtubules from MI to the MII stage of mouse oocytes. We costained Txndc9 and *α*-tubulin, and their signals were overlapped, which further confirmed the colocalization of Txndc9 and *α*-tubulin in mouse oocytes. Moreover, mouse Txndc9 contains the conserved ATP binding motif P-loop, similar to dynein heavy chain and other cytoskeletal proteins. Txndc9 is essential for proper microtubule organization and function, and cell division was disturbed after loss of Txndc9 in* C. elegans* [8]. In* Arabidopsis*, it also has been reported that Txndc9 is required for proper actin and tubulin function and plays a key role in establishing the functional cytoskeleton in cell division [6]. In mammals, oocytes accurate distribution of chromosomes requires intact meiotic spindle [27]. We asked whether Txndc9 could affect tubulin biogenesis during oocyte maturation. Our results showed that loss of Txndc9 disturbed the tubulin assembly and led to aberrant spindles morphologies in either immature or mature MII oocytes. These results were consistent with previous studies that Txndc9 participated in spindle assembly [6, 8]. It has been reported that human Txndc9, as a noval CCT-binding protein, has negative effects on actin and tubulin folding. Txndc9 and PFD activities help CCT and modulate the level of folded actin and tubulin, an essential process for maintenance of the functional cytoskeleton [10]. Whether Txndc9 regulates the level of folded tubulin by forming the ternary complex with tubulin and CCT during mouse oocyte maturation needs further research.

Disulfide bonds between the *α*-tubulin and the *β*-tubulin subunits were repaired by thioredoxin, and tubulin polymerization may be redox-regulated [16]. Txndc9 has atypical redox active domain, suggesting that Txndc9 may be required for the balance of redox during mouse oocyte maturation. Here, we detected the redox potential and antioxidant ability of mouse oocyte after loss of Txndc9. Our results showed a large proportion of GV stage oocytes depleted of Txndc9 subjected to oxidative stress response with an increase in ROS fluorescence and a decrease in MCB fluorescence.

ROS is generated in mitochondria and plays a key role in various physiological processes, such as oocyte maturation, gamete interaction, and embryo development [28, 29]. Level of ROS has been associated with the alteration of mitochondrial content [30]. Glutathione (GSH), a tripeptide thiol, has numerous biological functions in mammalian cells. GSH, an antioxidant molecule, acts as a defense mechanism in oxidative stress by maintaining the intracellular redox status [31]. GSH plays an important role in maintaining the meiotic spindle morphology of oocyte. Moreover, GSH resists the toxic activity of ROS by interacting with its associated enzymes [26]. The level of GSH is well documented and associated with oocyte quality during oocyte maturation [32, 33]. An imbalance between the concentrations of reactive oxygen species (ROS) and antioxidants, which results in oxidative damage of cell macromolecules, defined as oxidative stress affects oocytes quality and maturation to fertilization and embryo development [34]. And oxidative stress could affect microtubule dynamics and chromosomal alignments [35]. Our results suggested that Txndc9 is essential for maintaining mitochondrial content and balancing redox. We speculated that loss of Txndc9 disrupted redox balance, induced ROS overproduction, and decreased MCB level of oocytes that undergone oxidative stress and then further disrupted oocytes spindle assembly and impaired maturation. It has been reported that the formation and repair of tubulin disulfide and microtubule assembly are regulated by oxidation of key cysteine residues by reactive oxygen species (ROS)/thioredoxin reductase system [16, 17]. We hypothesized that oocyte depleted of Txndc9 subjected to abnormal tubulin assembly is related to higher level of ROS. It needs further study to confirm this hypothesis.

Taken together, our results indicated that Txndc9 is an important protein during the maturation of mouse oocyte. It is required for spindle assembly and redox balance during oocyte meiosis.
